# Effects of remimazolam on postanesthetic shivering and related clinical responses in patients undergoing cesarean section

**DOI:** 10.3389/fmed.2026.1776678

**Published:** 2026-03-27

**Authors:** Ziyi Zhang, Minghong Liu, Qiang Zhang, Qingyu Wang, Liang Zhang, Hui Zhao, Yufeng Wang, Lili Li, Wenzhe Shen, Jun Shi

**Affiliations:** 1 Graduate School, Bengbu Medical University, Bengbu, China; 2 Department of Anesthesiology, The First Affiliate Hospital of Anhui University of Science and Technology, Huainan, China

**Keywords:** cesarean section, core body temperature, remimazolam, shivering, spinal anesthesia

## Abstract

**Purpose:**

This study aimed to evaluate the association between remimazolam administration and perioperative shivering as well as related clinical outcomes in patients undergoing cesarean section under neuraxial anesthesia.

**Methods:**

A total of 120 patients undergoing cesarean section under neuraxial anesthesia were randomly allocated to a remimazolam group (R), a remimazolam plus flumazenil group (RF), or a normal saline group (C) (*n* = 40 each). In the R group, remimazolam was administered intravenously at 0.1 mg/kg after fetal delivery, followed by continuous infusion at 0.2 mg/kg/h until the end of surgery. In the RF group, flumazenil was administered at the end of surgery to reverse sedation. The C group received an equivalent volume of normal saline. The incidence and severity of perioperative shivering, changes in perioperative body temperature, intraoperative traction response scores, and perioperative adverse events were compared among the three groups.

**Results:**

The incidence of perioperative shivering was 17.5% in the R group and 30.0% in the RF group, both of which were significantly lower than that in the C group (52.5%; *p* < 0.05). Intraoperative and postoperative body temperatures were higher in the R group than in the C group (*p* < 0.05). During uterine cavity manipulation, traction response scores were lower in the R and RF groups than in the C group (*p* < 0.05). The incidence of postoperative nausea and vomiting was lower in the R and RF groups than in the C group (*p* < 0.05).

**Conclusion:**

In patients undergoing cesarean section under neuraxial anesthesia, remimazolam administration after fetal delivery was associated with a lower incidence of perioperative shivering, higher perioperative body temperature, and reduced intraoperative traction responses. Differences in perioperative adverse events were observed among the groups. The reduction in perioperative shivering was partially preserved after reversal with flumazenil.

## 1 Introduction

Perioperative shivering is a common anesthesia-related complication, with a particularly high incidence during neuraxial anesthesia and in patients experiencing hypothermia-related interventions (1–4). Shivering markedly increases metabolic rate and oxygen consumption and may lead to excessive activation of the sympathetic nervous system, thereby increasing the risk of adverse events such as myocardial ischemia and respiratory impairment (5–8). Previous studies have reported that the incidence of shivering in parturients undergoing cesarean section under neuraxial anesthesia can exceed 50% (9). Sympathetic blockade induced by neuraxial anesthesia leads to peripheral vasodilation and redistribution of body heat, which are key mechanisms contributing to perioperative hypothermia and shivering in patients undergoing cesarean section (10). In addition, traction and manipulation of the uterus and the abdominal organs during surgery may elicit pronounced traction responses, which manifest as pain, discomfort, nausea, vomiting, and anxiety. These factors can also trigger non-thermoregulatory shivering via the central nervous system pathways (11). Therefore, effectively preventing shivering and improving intraoperative comfort while ensuring maternal and neonatal safety remains a significant challenge in obstetric anesthesia. Remimazolam is a novel ultra-short-acting benzodiazepine sedative characterized by rapid onset, fast metabolism, and minimal effects on respiratory and hemodynamic function (12–14). In recent years, the safety and efficacy of remimazolam have been increasingly demonstrated in procedural sedation and general anesthesia. However, evidence regarding the use of remimazolam for the prevention of perioperative shivering and attenuation of intraoperative traction responses in patients undergoing cesarean section under neuraxial anesthesia remains limited. In particular, systematic clinical data are lacking on its effects on shivering incidence and perioperative body temperature when administered after fetal delivery. Based on this background, the present study aimed to investigate the effects of remimazolam administered after fetal delivery on the incidence of perioperative shivering, changes in perioperative body temperature, and intraoperative traction responses in patients undergoing cesarean section under neuraxial anesthesia, with a preliminary exploration of the underlying mechanisms. We hypothesized that continuous infusion of remimazolam after fetal delivery would modulate thermoregulatory responses and reduce the incidence of shivering. The findings of this study may provide additional clinical evidence for pharmacological strategies to prevent perioperative shivering in patients undergoing cesarean section.

## 2 Materials and methods

### 2.1 General information

A total of 120 patients undergoing cesarean section under neuraxial anesthesia at the First Affiliated Hospital of Anhui University of Science and Technology between August and November 2025 were enrolled in this study. The study was approved by the Institutional Ethics Committee (No. 2025-KY-Y003-001), and written informed consent was obtained from all patients or their legal representatives. The trial was registered with the Chinese Clinical Trial Registry on 14 July 2025 (No. ChiCTR2500105881). The exclusion criteria included refusal to participate, pregnancy-induced hypertension, preeclampsia or eclampsia, multiple pregnancy, contraindications to neuraxial anesthesia, or contraindications to remimazolam. The withdrawal criteria included a tympanic temperature on admission higher than 37.0 °C or lower than 36.0 °C, an insufficient sensory block level below T6, conversion to general anesthesia during surgery, an intraoperative Ramsay sedation score greater than 3, intraoperative blood loss exceeding 600 mL, and loss to follow-up after surgery. Patients were randomly assigned using a random number table into three groups: the remimazolam group (R group, *n* = 40), the remimazolam plus flumazenil group (RF group, *n* = 40), and the normal saline group (C group, *n* = 40).

### 2.2 Anesthesia methods

Patients fasted for at least 8 h for solids and 2 h for liquids. After the patient entered the operating room, venous access was established, and 250 mL of 6% hydroxyethyl starch was rapidly infused. Standard monitoring included electrocardiography, mean arterial pressure (MAP), heart rate (HR), and pulse oxygen saturation (SpO_2_). Tympanic temperature was measured using an infrared ear thermometer gently inserted into the external auditory canal. Core temperature was measured using a flexible rectal temperature probe connected to a GE monitor, which was covered with a disposable sheath, lubricated with lidocaine cream, gently inserted 5 cm into the rectum, and secured to the inner thigh. Combined spinal-epidural anesthesia was administered at the L2–L3 interspace. Intrathecally, 15 mg of 0.5% ropivacaine was injected, followed by epidural catheter placement 3 cm into the epidural space. The operating table was tilted 15° to the left to prevent hypotension, and the sensory block level was maintained between T6 and T8. After fetal delivery and umbilical cord clamping, patients in the R group received intravenous remimazolam at 0.1 mg/kg, followed by continuous infusion at 0.2 mg/kg/h until the end of surgery. At the end of surgery, 5 mL of normal saline was administered. In the RF group, patients received flumazenil 0.2 mg at the end of surgery in addition to the regimen used in the R group (diluted in 5 mL of normal saline and administered by slow intravenous injection). If the Ramsay Sedation Score remained >2 within 60 s, an additional 0.1 mg was administered and repeated as needed until adequate awakening was achieved (Ramsay score ≤2), with a maximum total dose of 0.4 mg. The C group received an equivalent volume of normal saline at corresponding time points. Sedation in the R and RF groups was assessed using the Ramsay Sedation Scale and maintained at scores of 2–3. Postoperative analgesia was provided via patient-controlled intravenous analgesia (PCIA) containing 0.1 mg of sufentanil and 30 mg of azasetron diluted to 100 mL, with a background infusion of 2 mL/h, a bolus dose of 1 mL, and a lockout interval of 10 min. Patients were transferred to the postanesthesia care unit (PACU) for 30 min and returned to the ward when the Aldrete score exceeded 8. Shivering was assessed every 15 min. Tramadol at a dose of 0.5 mg/kg was administered intravenously if the Wrench score was ≥2 for ≥5 min. A decrease in body temperature greater than 0.5 °C was defined as hypothermia. Respiratory depression was defined as an oxygen saturation (SpO_2_) below 90% and was managed with a jaw-thrust maneuver and supplemental oxygen via a face mask. Hypotension (>25% decrease from the baseline) was treated with 6 mg of ephedrine. Bradycardia (HR < 50 beats/min) was treated with atropine if necessary. Temperatures in the operating room and PACU were maintained at 23 ± 0.5 °C, with a humidity range of 55–65%. Duration of surgery, fluid administration, urine output, and blood loss were recorded.

### 2.3 Observation indicators

Shivering was assessed using the Wrench shivering scale every 15 min from fetal delivery until 1 h after the end of surgery. The Wrench shivering scale was defined as follows: 0, no shivering (no visible muscle activity); 1, mild shivering (piloerection, peripheral vasoconstriction, pallor, or cyanosis without visible muscle activity); 2, mild muscular activity involving one muscle group; 3, moderate shivering involving more than one muscle group; and 4, severe, generalized shivering involving the whole body. The distribution of shivering severity grades was recorded. The overall incidence of shivering was calculated as the proportion of patients with Wrench score ≥1. Rectal and tympanic temperatures were recorded on admission to the operating room (T0), before fetal delivery (T1), 10 min after drug administration (T2), 20 min after drug administration (T3), and at the end of surgery (T4). Tympanic temperature was additionally recorded on arrival at the postanesthesia care unit (PACU; T5) and at discharge from the PACU (T6). The severity of traction response was assessed during fetal delivery (E1) and uterine cavity cleaning after placental delivery (E2). Each assessment was performed within 60 s of traction using the Observer’s Assessment of Response to Traction (OART) Scale. The OART scores were defined as follows: 0, no response (calm, no movement, facial expression change, or vocalization); 1, mild response (slight frowning, mild discomfort, or soft groaning without limb movement); 2, moderate response (obvious discomfort, vocalization such as crying out, and/or localized movement, e.g., lifting the arm or the head); and 3, severe response (marked body movement such as arching or attempting to sit up, with or without nausea, retching, or vomiting, interfering with surgical procedures). The mean arterial pressure (MAP) and heart rate (HR) were recorded at each predefined time point. The proportion of patients requiring tramadol for the management of shivering and the administered doses were recorded. Adverse events, including hypotension, respiratory depression, postoperative nausea and vomiting, and cardiac arrhythmia, were recorded and compared among the groups.

### 2.4 Randomization and blinding

This study was designed as a prospective, randomized, double-blind, controlled trial. A total of 120 eligible patients were randomly assigned in a 1:1:1 ratio to the remimazolam group (R), the remimazolam plus flumazenil group (RF), and the control group (C). The randomization sequence was generated by an independent physician using SPSS software and concealed in sequentially numbered, identical, opaque, sealed envelopes. After the patient entered the operating room, an independent physician not involved in the study opened the envelope, determined the allocation, and prepared the corresponding medication. Patients, the anesthesiologist administering anesthesia, and the data collector were all blinded to group assignments. The data collector was responsible solely for assessing and recording physiological variables and was not aware of the study protocol.

### 2.5 Statistical methods

The sample size was calculated using PASS 15.0 software. Based on previous studies, the incidence of shivering was assumed to be 40, 15, and 30% in the C, R, and RF groups, respectively. With *α* = 0.05 and a power (1 − *β*) of 0.80, a total sample size of 96 patients was required. Considering a 10% dropout rate, 120 patients were enrolled. Data were analyzed using SPSS 27.0. Continuous variables with a normal distribution were expressed as mean ± standard deviation (SD). Comparisons of continuous variables among multiple groups were performed using a one-way analysis of variance (ANOVA), followed by *post-hoc* multiple comparisons using the Student–Newman–Keuls (SNK) test. Ordinal data were compared using the Kruskal–Wallis *H* test, and when a significant difference was detected, *post-hoc* pairwise comparisons were conducted using Dunn’s test with Bonferroni correction. Categorical variables were analyzed using the chi-squared test for multiple proportions. For rare events with small sample sizes, the Monte Carlo exact test was used.

## 3 Results

### 3.1 Basic situation comparison

There were no significant differences among the three groups in age, body mass index, baseline body temperature (T0), intraoperative blood loss, fluid administration, urine output, or duration of surgery (Table 1).

**Table 1:** Comparison of general information and baseline characteristics among the three groups.

General information	R group	RF group	C group	*p*-value
Age (years)	30.07 ± 4.56	30.98 ± 5.10	30.90 ± 4.96	0.6587
Body mass index (BMI) (kg/m^2^)	28.78 ± 2.88	28.30 ± 3.19	28.64 ± 2.39	0.7375
Basal body temperature (°C)	36.72 ± 0.15	36.69 ± 0.14	36.71 ± 0.12	0.6845
Blood loss (ml)	307.75 ± 83.00	320.50 ± 83.11	301.25 ± 85.10	0.7302
Infusion volume (ml)	716.25 ± 157.86	671.25 ± 176.46	682.00 ± 174.86	0.4677
Urine output (ml)	236.25 ± 76.52	253.25 ± 76.94	245.00 ± 81.62	0.6259
Surgery time (min)	36.72 ± 4.23	38.00 ± 7.19	37.12 ± 5.42	0.5986

### 3.2 Comparison of cold shivering reactions

Table 2 summarizes the comparison of shivering responses among the groups. The distribution of shivering severity differed significantly among the three groups (Kruskal–Wallis *H* = 11.14, *p* = 0.004). *Post-hoc* analysis using Dunn’s test and Bonferroni correction revealed that shivering severity was significantly lower in the R group than in the C group (adjusted *p* = 0.0045). No significant differences were observed between the R group and the RF group or between the RF group and the C group after Bonferroni correction (both adjusted *p* > 0.05). The incidence of shivering was lowest in the R group (17.5%), followed by the RF group (30.0%), and highest in the C group (52.5%).

**Table 2:** Comparison of shivering severity and incidence among the three groups.

Group	*n*	Grade 0	Grade 1	Grade 2	Grade 3	Grade 4	Incidence (%)
R	40	33	4	2	1	0	17.5
RF	40	28	8	3	1	0	30
C	40	19	12	6	3	0	52.5
*p*-value	–	0.004					0.003

### 3.3 Core body temperature comparison

**Figure 1:**
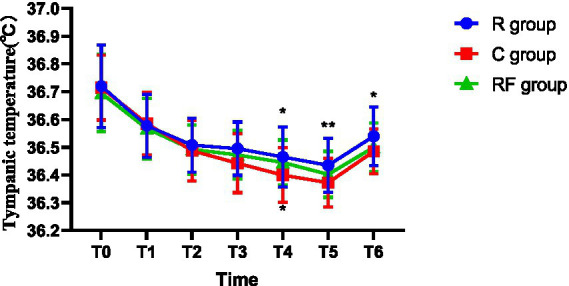
Changes in tympanic temperature at different time points among the three groups.

**Figure 2:**
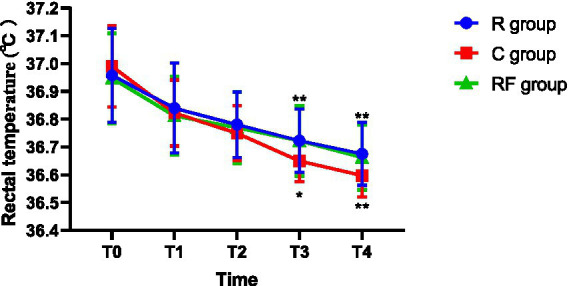
Changes in rectal temperature at different time points among the three groups.

Body temperature measurements, including tympanic and rectal temperatures, are summarized in Table 3 (Figure 1) and Table 4 (Figure 2). Tympanic temperature did not differ significantly among the groups at baseline (T0) or during the early intraoperative period (T1–T2). At T3, a significant difference in rectal temperature was observed among the three groups, with higher temperatures in the remimazolam-related groups compared with the control group. No significant difference was detected in the overall comparison of tympanic temperature. However, a pairwise analysis revealed that the tympanic temperature in the R group was significantly higher than that in the C group. Significant intergroup differences in tympanic temperature were observed at T4 and T5, with higher temperatures in the R group and, at T4, also in the RF group compared with the C group. At T6, tympanic temperature remained higher in the R group than in the C group. Rectal temperature was comparable among the groups at T0, T1, and T2; significant differences emerged at later time points. At both T3 and T4, rectal temperature was significantly higher in the R and RF groups than in the C group.

**Table 3:** Comparison of tympanic temperature at different time points among the three groups.

Time point	R group (°C)	RF group (°C)	C group (°C)	*p*-value
T0	36.72 ± 0.15	36.69 ± 0.14	36.71 ± 0.12	0.6845
T1	36.57 ± 0.11	36.56 ± 0.11	36.58 ± 0.11	0.7825
T2	36.50 ± 0.10	36.49 ± 0.09	36.48 ± 0.11	0.6417
T3	36.49 ± 0.10^*^	36.47 ± 0.09	36.44 ± 0.11	0.0556
T4	36.46 ± 0.11^*^	36.44 ± 0.08^*^	36.40 ± 0.10	0.0103
T5	36.43 ± 0.10^**^	36.40 ± 0.08	36.37 ± 0.09	0.0094
T6	36.54 ± 0.11^*^	36.50 ± 0.09	36.48 ± 0.08	0.0244

Comparisons among the three groups were performed using a one-way analysis of variance (ANOVA). Pairwise comparisons were conducted between the groups using the Student–Newman–Keuls (SNK) *q* test according to the prespecified analysis plan, regardless of the significance of the overall ANOVA. ^*^p < 0.05 and ^**^p < 0.01 indicate comparisons with the C group.

**Table 4:** Comparison of rectal temperature at different time points among the three groups (°C, *x* ± *s*).

Time point	R group	RF group	C group	*p*-value
T0	36.95 ± 0.17	36.94 ± 0.16	36.99 ± 0.15	0.3955
T1	36.84 ± 0.16	36.81 ± 0.14	36.82 ± 0.12	0.6807
T2	36.78 ± 0.12	36.77 ± 0.13	36.74 ± 0.10	0.4351
T3	36.72 ± 0.11^**^	36.72 ± 0.13^**^	36.64 ± 0.07	0.0024
T4	36.67 ± 0.11^**^	36.66 ± 0.12^**^	36.59 ± 0.07	0.0008

Comparisons among the three groups were performed using a one-way analysis of variance (ANOVA). Pairwise comparisons were conducted between the groups using the Student–Newman–Keuls (SNK) *q* test according to the prespecified analysis plan, regardless of the significance of the overall ANOVA. ^**^p < 0.01 indicates comparisons with the C group.

### 3.4 Tendon reflexes comparison

**Figure 3:**
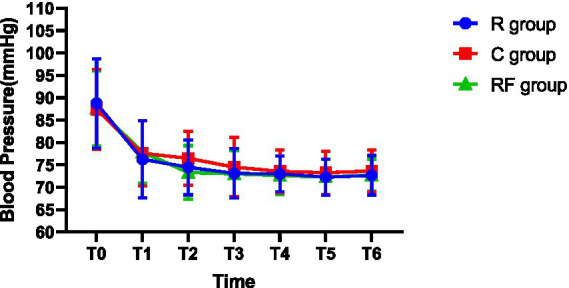
Changes in the mean arterial pressure at different time points among the three groups.

**Figure 4:**
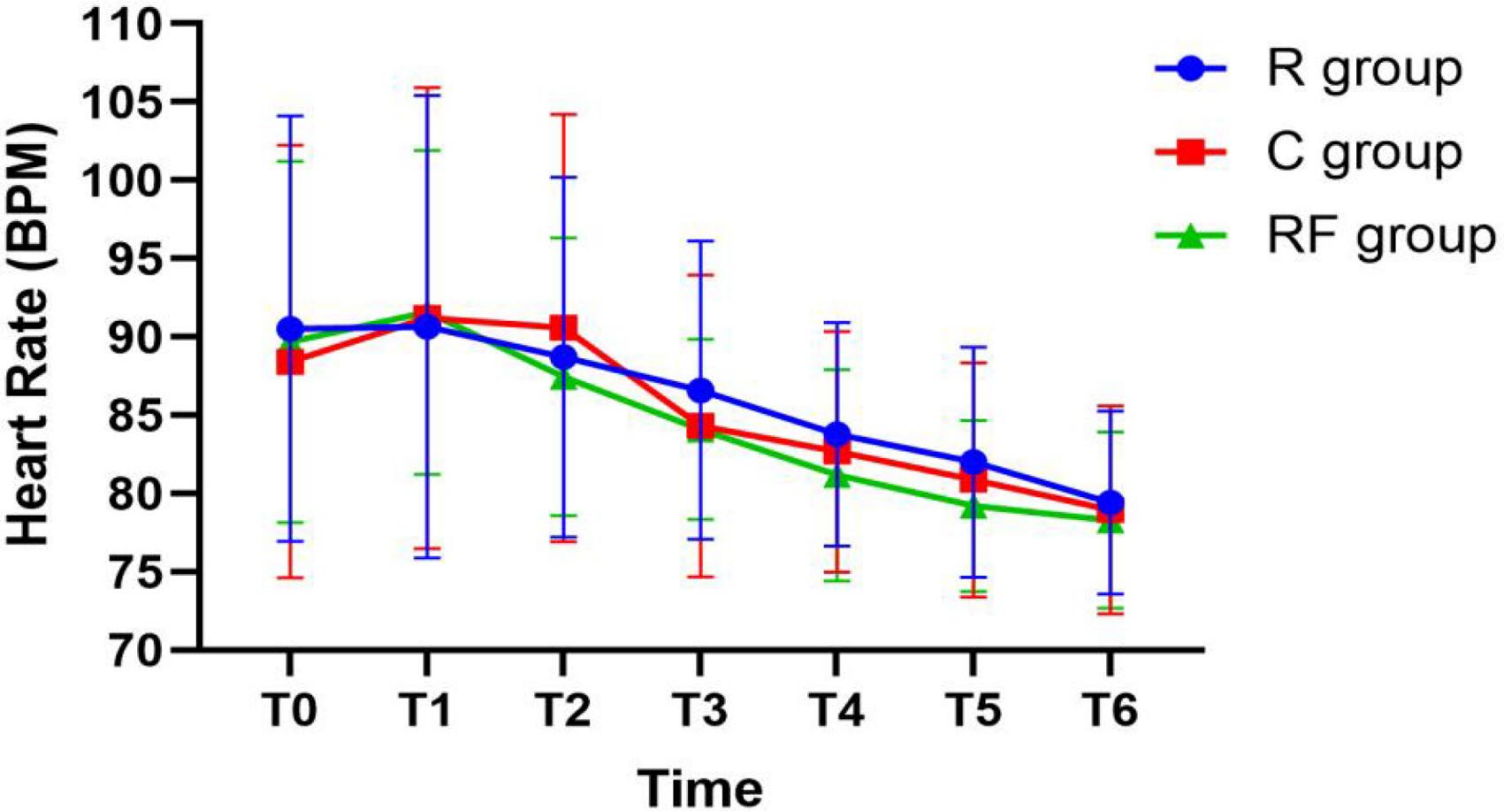
Changes in heart rate at different time points among the three groups.

Table 5 (Figure 3) and Table 6 (Figure 4) present the traction responses of the groups at the E1 and E2 time points, respectively. At the E1 time point, the distribution of traction response severity did not differ significantly among the three groups. No significant differences were observed in the proportions of no, mild, moderate, or severe responses (all *p* > 0.05). The overall incidence of traction response was comparable among the R, RF, and C groups. At the E2 time point, the distribution of traction response severity differed significantly among the groups. Compared with the C group, patients in the R and RF groups exhibited a lower incidence and milder severity of traction responses. Specifically, the proportions of no response and mild response were higher in the R and RF groups, whereas moderate responses were observed only in the C group.

**Table 5:** Comparison of traction response among the three groups at E1.

Group	*n*	No response	Mild response	Moderate response	Severe response	Incidence (%)
R	40	9	19	10	2	77.5
RF	40	11	17	9	3	72.5
C	40	10	20	8	2	75
*p*-value	–	0.91				0.875

**Table 6:** Comparison of traction response among the three groups at E2.

Group	*n*	No response	Mild response	Moderate response	Severe response	Incidence (%)
R	40	29	11	0	0	30
RF	40	30	10	0	0	25
C	40	19	19	2	0	50
*p*-value	–	0.012				0.017

### 3.5 Hemodynamic comparison

Hemodynamic parameters are presented in Tables 7 and 8. Mean arterial pressure and heart rate were comparable among the groups at baseline and throughout the intraoperative period, with no significant intergroup differences observed at any time point (all *p* > 0.05).

**Table 7:** Changes in the mean arterial pressure at different time points (mmHg, *x* ± *s*).

Time point	R group	RF group	C group	*p*-value
T0	88.72 ± 9.96	87.62 ± 8.37	87.45 ± 8.88	0.7940
T1	76.27 ± 8.64	77.90 ± 7.02	77.60 ± 7.34	0.5176
T2	74.47 ± 6.15	73.30 ± 6.00	76.47 ± 6.03	0.0645
T3	73.10 ± 5.85	73.02 ± 5.21	74.50 ± 6.63	0.4434
T4	72.57 ± 3.63	72.65 ± 4.31	73.57 ± 4.79	0.5086
T5	72.25 ± 4.06	72.30 ± 3.92	73.25 ± 4.81	0.5020
T6	72.67 ± 4.37	72.72 ± 3.64	73.65 ± 4.66	0.5137

**Table 8:** Changes in heart rate at different time points (bpm, *x* ± *s*).

Time point	R group	RF group	C group	*p*-value
T0	90.52 ± 13.59	89.67 ± 11.54	88.42 ± 13.81	0.7690
T1	90.65 ± 14.75	91.58 ± 10.35	91.20 ± 14.71	0.9531
T2	88.70 ± 11.48	87.45 ± 8.88	90.57 ± 13.65	0.4756
T3	86.60 ± 9.52	84.10 ± 5.74	84.32 ± 9.64	0.3496
T4	83.77 ± 7.14	81.17 ± 6.75	82.67 ± 7.69	0.2727
T5	82.00 ± 7.36	79.20 ± 5.46	80.87 ± 7.49	0.1871
T6	79.42 ± 5.84	78.30 ± 5.61	78.95 ± 6.65	0.7064

### 3.6 Tramadol intervention status

The number of patients requiring tramadol intervention and the administered doses did not differ significantly among the groups (Table 9).

**Table 9:** Comparison of the number of patients requiring tramadol intervention and tramadol dosage among the three groups.

Group	*n*	Tramadol intervention	Tramadol dosage (mg, $\bar{x}$ ± *s*)
R	40	3	36.66 ± 3.05
RF	40	3	38.33 ± 3.51
C	40	6	38.16 ± 4.07
*p*-value	–	0.5895	0.8253

### 3.7 Adverse reaction status

The incidence of adverse events is summarized in Table 10. No significant differences were observed among the three groups in the incidence of hypotension, hypothermia, respiratory depression, or arrhythmia. The incidence of nausea and vomiting differed significantly among the groups, with the highest incidence observed in the C group.

**Table 10:** Comparison of adverse events among the three groups.

Adverse event	R group (*n* = 40)	RF group (*n* = 40)	C group (*n* = 40)	*p*-value
Hypotension	10(25%)	9 (22.5%)	6 (15.0%)	0.5185
Hypothermia	2 (5.0%)	3 (7.5%)	6 (15.0%)	0.3782
Respiratory depression	3 (7.5%)	2 (5.0%)	0 (0%)	0.3681
Nausea and vomiting	3 (7.5%)	4 (10.0%)	11 (27.5%)	0.0241
Arrhythmia	2 (5.0%)	3 (7.5%)	4 (10.0%)	0.9078

## 4 Discussion

The present study focused on perioperative shivering in patients undergoing cesarean section under neuraxial anesthesia and evaluated the association between remimazolam administration after fetal delivery and shivering-related clinical outcomes. The results indicated that, compared with the control group, patients receiving remimazolam had a lower incidence of perioperative shivering and reduced intraoperative traction responses. These findings suggest that, under the specific timing of administration and anesthetic conditions applied in this study, remimazolam administration was associated with improved perioperative clinical responses in patients undergoing cesarean section.

The incidence of shivering is relatively high in patients undergoing cesarean section under neuraxial anesthesia, and its pathogenesis is multifactorial. Neuraxial anesthesia induces sympathetic blockade, leading to peripheral vasodilation and redistribution of body heat, which is a key mechanism underlying perioperative hypothermia (15). In addition, surgical exposure, a cold operating room environment, and the infusion of cold fluids contribute to increased perioperative heat loss, collectively promoting the onset of shivering (16).

Traction of the uterus and abdominal organs, surgical stress, and perioperative anxiety may also trigger shivering via the central nervous system pathways (17, 18). Thus, shivering is not solely determined by changes in body temperature but results from the combined effects of thermoregulatory and non-thermoregulatory mechanisms. In this study, we prospectively controlled the main external factors influencing perioperative hypothermia and shivering. The temperature and humidity of the operating room were standardized, and the surgery duration, intravenous fluid volume, blood loss, and urine output were recorded. A consistent spinal anesthesia protocol was also applied to minimize environmental confounding factors. The results of this study showed that intraoperative body temperature decreased to varying degrees in all three groups; however, the magnitude of temperature decline in the R and RF groups was significantly smaller than that in the C group. After transfer to the PACU, body temperature increased in all groups; however, tympanic temperature in the R group remained higher than that in the C group at T5 and T6. At T3, discrepancies were observed among the three groups between rectal and tympanic temperatures, which may be related to the physiological characteristics of the measurement sites and their response lag. Rectal temperature more accurately reflects core temperature but responds slowly to rapid temperature changes, whereas tympanic temperature may reflect early peripheral heat redistribution but is more susceptible to environmental influences such as facial cooling, sweating, and airflow. Although there were some differences in the trends of temperature change among the three groups, the incidence of shivering was not entirely consistent with the changes in body temperatures. This finding suggests that shivering in patients undergoing cesarean section under neuraxial anesthesia may be influenced not only by hypothermia but also by central regulatory mechanisms and tolerance to surgical stimuli.

Although shivering is a physiological thermoregulatory defense response aimed at restoring core temperature, its clinical significance depends on its severity. Mild shivering may help generate heat, whereas moderate to severe shivering significantly increases metabolic rate, oxygen consumption, and sympathetic activation. For women undergoing cesarean section, particularly those with limited cardiopulmonary reserve, excessive shivering may lead to unnecessary physiological stress and pose potential risks (19). Therefore, clinical management should not focus on eliminating all thermoregulatory responses but rather on suppressing clinically significant shivering that may affect maternal comfort and safety. In this study, tramadol was administered as rescue therapy only when shivering reached moderate to severe intensity, which is consistent with the treatment principles recommended in clinical guidelines (20, 21). Tramadol inhibits the reuptake of serotonin (5-HT) and norepinephrine (NE) in the central nervous system, thereby suppressing motor output signals from the brain to the spinal cord and directly blocking the reflex arc that causes muscle shivering. It also activates the *μ*-opioid receptor, affecting neuronal activity in the preoptic area of the hypothalamus (PO/AH), inhibiting the release of excitatory neurotransmitters, and reducing neuronal sensitivity to cold stimuli. These two mechanisms work synergistically to suppress shivering (22, 23). The anti-shivering mechanism of tramadol does not involve the Gamma-amin Obutyric Acid (GABA-A) receptor, and it has a mild respiratory suppressant effect. Additionally, tramadol has analgesic properties that help alleviate postoperative pain (24). However, it is associated with adverse effects, such as nausea, vomiting, and a reduced seizure threshold, related to its monoaminergic activity (25). From the perspective of neural pathways, tramadol mainly suppresses descending pathways and blocks the efferent transmission of the shivering reflex. In contrast, benzodiazepines primarily act on the hypothalamic thermoregulatory center, thereby lowering the shivering threshold.

As a novel ultra-short-acting benzodiazepine, remimazolam has attracted increasing clinical attention not only for its sedative and anxiolytic properties but also for its potential anti-shivering effects. The underlying mechanisms may involve central regulation, neural transmission, and receptor-mediated pathways (26, 27). From the perspective of central regulation, shivering is a thermogenic defense response initiated by the hypothalamic thermoregulatory center in response to a decrease in core body temperature (28, 29). Remimazolam crosses the blood–brain barrier and interacts with GABA-A receptors in the hypothalamus, thereby enhancing GABA-mediated inhibitory neurotransmission. This effect may suppress shivering-related signaling from the thermoregulatory center and attenuate the reflex pathway linking core temperature reduction to the initiation of shivering (30). In addition, the sedative effects of remimazolam may reduce central sensitivity and diminish the perception of cold stimuli, indirectly lowering the likelihood of shivering initiation. Along neural transmission pathways, shivering signals are conveyed to skeletal muscles through the anterior horn motor neurons of the spinal cord, resulting in rhythmic muscular contractions. Remimazolam may also act on GABA-A receptors at the spinal level, inhibiting the transmission of shivering-related neural impulses and reducing motor neuron excitability, thereby decreasing the frequency and intensity of skeletal muscle contractions (31). Furthermore, remimazolam may alleviate shivering by attenuating excessive sympathetic activation, thereby reducing peripheral vasoconstriction and muscle tension (32, 33). Notably, the sedative state itself may have contributed substantially to the findings of the present study. In this study, patients in the remimazolam-related groups were maintained at a light level of sedation, whereas those in the control group did not receive sedative intervention. Sedation per se may reduce the risk of non-thermoregulatory shivering by alleviating anxiety and decreasing central nervous system excitability.

Flumazenil is a selective antagonist of benzodiazepine receptors that competitively binds to these receptors and reverses the central inhibitory effects of remimazolam (34, 35). An animal study demonstrated that remimazolam attenuates changes in core body temperature during shivering in rabbits, potentially suppressing shivering through the GABAergic pathway and that this anti-shivering effect can be antagonized by the GABA-A receptor antagonist flumazenil (26). In the present study, patients in the remimazolam plus flumazenil group exhibited a partial change in shivering incidence following antagonism, but shivering did not fully return to the level observed in the control group. These findings suggest that the anti-shivering effect of remimazolam may not be entirely dependent on sedative effects that are immediately reversible and may also be related to alterations in central thermoregulatory control. Although flumazenil antagonized part of the effects of remimazolam, it is possible that remimazolam had already exerted an influence on thermoregulation and shivering thresholds during the surgical period. Prior to the end of surgery, remimazolam may have stabilized hypothalamic thermoregulatory function and increased the shivering threshold, effects that may have partially persisted after flumazenil administration. In addition, antagonism by flumazenil is a gradual process, during which residual effects of remimazolam may persist, potentially contributing to the continued lower incidence of shivering. However, as plasma concentrations of remimazolam and flumazenil were not measured, the extent of antagonism could not be precisely determined. Therefore, these interpretations are based solely on clinical observations and cannot be regarded as direct evidence of the underlying mechanisms. Consequently, the findings related to flumazenil should be considered hypothesis-generating rather than confirmatory with respect to the anti-shivering mechanisms of remimazolam.

Another notable characteristic of remimazolam is its favorable hemodynamic profile, as reported in previous studies (36). In the present study, intraoperative administration of remimazolam had minimal effects on blood pressure and heart rate. In terms of adverse events, the incidences of intraoperative and postoperative nausea and vomiting (IONV) were lower in the remimazolam and remimazolam plus flumazenil groups. One important contributing factor to nausea and vomiting may be sympathetic blockade induced by neuraxial anesthesia, which can lead to hypotension and subsequent activation of the vomiting center in the brainstem (37, 38). A study comparing remimazolam and midazolam for the prevention of nausea and vomiting during cesarean section showed that remimazolam significantly reduced both the incidence and severity of IONV, possibly owing to its lower incidence of hypotension (39). In contrast, no significant differences in the incidence of hypotension were observed among the three groups in the present study. Therefore, the reduced incidence of nausea and vomiting associated with remimazolam may be indirectly related to its sedative effects and attenuation of intraoperative traction responses rather than to hemodynamic changes alone.

Several limitations of the present study should be acknowledged. First, the confounding effect of sedation depth was not fully excluded. The independent pharmacological anti-shivering effect of remimazolam could not be completely distinguished from the effect of sedation itself. Differences in the depth of sedation may have a significant impact on shivering and traction responses. As no positive control was included and the study focused only on the comparison between the remimazolam and saline groups, it was difficult to precisely determine whether the observed effects were attributable to the drug’s specific pharmacological action or were indirectly related to differences in sedation depth, which may have interfered with the causal interpretation of the study findings. Second, the relatively small sample size may limit the generalizability of the findings. In addition, this study focused primarily on the incidence of shivering, while the molecular mechanisms by which remimazolam may influence thermoregulation and shivering were not investigated. Future investigations incorporating molecular and cellular approaches may help to further elucidate the mechanisms underlying the effects of remimazolam and provide stronger theoretical support for its clinical use. Finally, remimazolam concentrations in breast milk were not measured, and, therefore, its safety profile during breastfeeding could not be determined.

## 5 Conclusion

The present study suggests that administration of remimazolam after fetal delivery in patients undergoing cesarean section under neuraxial anesthesia may be associated with a reduced incidence of perioperative shivering and improved intraoperative comfort. These findings provide preliminary evidence supporting remimazolam as a potential pharmacological option for the prevention of shivering in this patient population. However, until its mechanisms are further clarified and study designs are optimized in future research, the clinical application of remimazolam should be considered with caution and individualized to patient circumstances.

## Data Availability

The datasets presented in this study can be found in online repositories. The names of the repository/repositories and accession number(s) can be found in the article/supplementary material.
